# Dual feature-based and example-based explanation methods

**DOI:** 10.3389/frai.2025.1506074

**Published:** 2025-02-10

**Authors:** Andrei Konstantinov, Boris Kozlov, Stanislav Kirpichenko, Lev Utkin, Vladimir Muliukha

**Affiliations:** Department of Artificial Intelligence Technologies, Peter the Great St. Petersburg Polytechnic University, St. Petersburg, Russia

**Keywords:** machine learning, explainable AI, neural additive network, dual representation, convex hull, example-based explanation, feature-based explanation

## Abstract

A new approach to the local and global explanation based on selecting a convex hull constructed for the finite number of points around an explained instance is proposed. The convex hull allows us to consider a dual representation of instances in the form of convex combinations of extreme points of a produced polytope. Instead of perturbing new instances in the Euclidean feature space, vectors of convex combination coefficients are uniformly generated from the unit simplex, and they form a new dual dataset. A dual linear surrogate model is trained on the dual dataset. The explanation feature importance values are computed by means of simple matrix calculations. The approach can be regarded as a modification of the well-known model LIME. The dual representation inherently allows us to get the example-based explanation. The neural additive model is also considered as a tool for implementing the example-based explanation approach. Many numerical experiments with real datasets are performed for studying the approach. A code of proposed algorithms is available. The proposed results are fundamental and can be used in various application areas. They do not involve specific human subjects and human data.

## 1 Introduction

Many machine learning models, including neural networks, have the black-box nature due to their complexity and the obscurity of their internal workings. Therefore, to explain how predictions are obtained for their corresponding inputs, specific explanation methods are required. This requirement affects many applications, especially those in medicine, finance, and safety maintenance. As a result, many successful methods and algorithms have been developed to satisfy this requirement (Arya et al., 2019; Belle and Papantonis, 2021; Guidotti et al., 2019; Liang et al., 2021; Molnar, 2019; Murdoch et al., 2019; Ras et al., 2022; Zablocki et al., 2021; Zhang Y. et al., 2021).

There are many definitions and interpretations of the explanation. We understand explanation as an answer to the question which features of an instance or a set of instances significantly impact the black-box model prediction or which features are most relevant to the prediction. Methods answering this question can be referred to as *feature importance* methods or the *feature-based explanation*. Another group of explanation methods is called the *example-based* explanation methods (Molnar, 2019). The corresponding methods are based on selecting influential instances from a training set having the largest impact on predictions to compare the training instance with the explainable one.

Feature importance explanation methods, in turn, can be divided into two groups: local and global. Methods from the first group explain the black-box model predictions locally around a test instance. Global methods explain a set of instances or the entire dataset. The well-known local explanation method is the Local Interpretable Model-Agnostic Explanation (LIME) (Ribeiro et al., 2016). In accordance with this method, a surrogate model is constructed and trained, which approximates the black-box model at a point. The surrogate model in LIME is the linear regression whose coefficients can be interpreted as the feature importance measures. In fact, LIME can be regarded as a method of the linear approximation of a complex non-linear function implemented by the black-box model at a point. LIME is based on using a simple regression model. Agarwal et al. (2021) proposed to generalize LIME using the generalized additive model (GAM) (Hastie and Tibshirani, 1990) instead of the simple linear regression and its implementation by means of neural networks of a special form. The GAM is a more general and flexible model in comparison with the original linear model. The corresponding surrogate model using the GAM is called the neural additive model (NAM).

Another important method, which is used for the local as well as global explanations, is SHapley Additive exPlanations (SHAP) (Lundberg and Lee, 2017; Strumbelj and Kononenko, 2010). The method is based on applying game-theoretic Shapley values (Shapley, 1953) which can be interpreted as average marginal contributions of features to the black-box model prediction. SHAP can be also viewed as a method of the linear approximation of the black-box model predictions.

**Figure 1:**
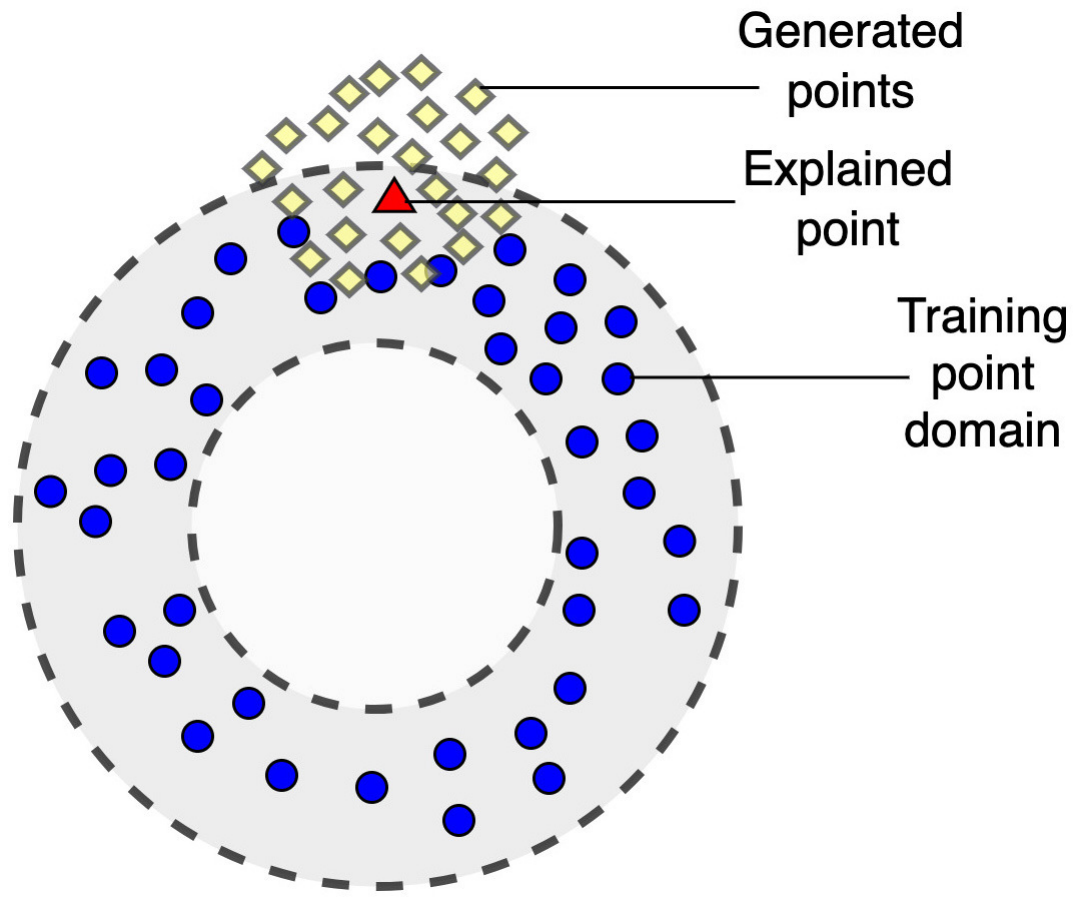
Illustration of a case of out-of-domain data when generated points may be out of the training point domain.

One of the important shortcomings of LIME is that it uses the perturbation technique which may be difficult to implement or may be even incorrect for some datasets, for example, for images. Moreover, it may provide incorrect results for high-dimensional data of a complex structure. The perturbation technique may generate a disturbed dataset especially when dealing with image data. A slight change in the data can lead to significant changes in images, often losing their meaning. Examples and an analysis of this pitfall as well as other pitfalls of LIME are considered in Molnar et al. (2020). The dual representation proposed in the study does not deal with images and allows us to overcome this difficulty. Another problem is that points generated in accordance with the perturbation technique may be located out of the training point domain, i.e., these points can be viewed as out-of-domain (OOD) data. This case is shown in Figure 1 where training points and generated points are depicted by small circles and by diamonds, respectively. The explained point is depicted by the triangle. A machine learning black-box model learned on points from the training domain may provide quite incorrect predictions for generated points which are outside of the domain. As a result, the approximating linear function constructed by using the generated points may be also incorrect.

One of the shortcomings of SHAP is that it is also computationally expensive when there is a large number of features due to considering all possible coalitions whose number is 2^*m*^, where *m* is the number of features. Therefore, the computational time grows exponentially. Several simplifications and approximations have been proposed to overcome this difficulty (Strumbelj and Kononenko, 2010, 2011, 2014; Utkin and Konstantinov, 2022). However, they do not cardinally solve the problem of high-dimensional data. Moreover, there is another difficulty of SHAP, which is rarely mentioned. According to SHAP, the black-box model prediction is computed for instances composed from subsets of features and some values of removing features introduced by using some rules. If to use the example depicted at Figure 1, then new instances in SHAP may be located inside or outside the ring bounding the training data domain where the black-box model provides incorrect predictions.

To partially solve the above problems, we propose a new explanation method which is based on applying two approaches: the *convex hull* of training data and the *duality* concept. The convex hull machine learning methods (Yousefzadeh, 2020) analyze relationship between a convex hull of a training set and the decision boundaries for test instances. The duality is a fundamental concept in various field. We use the dual representation of data assuming the linear space in the local area around the explainable instance.

The idea behind the proposed method is very simple. We propose to find the convex hull of a subset of training data consisting on *K* instances which are close to the explainable instance. By using extreme points of the corresponding convex polytope, each point inside the convex hull can be expressed through the linear combination of the extreme points. Coefficients λ of the linear combination are proposed to be regarded as a new feature vector which determines the corresponding point. They can be viewed as probabilities defined in the unit simplex of probabilities. Since the coefficients belong to the unit simplex, then they can be uniformly generated from the simplex such that each dual feature vector λ corresponds to the feature vector in the Euclidean space (the feature space of training data). A generated feature vector in the Euclidean space is computed through extreme points of the convex hull. As a result, we get a new dual dataset which generates instances in a local area around the explainable instance. The surrogate linear model is constructed by using this new dual dataset whose elements may have a smaller dimension defined by *K* or by the number of extreme points of the convex hull. Hence, we get important elements of the generated vectors of coefficients. Due to the linear representation of the surrogate (explanation) model, the important features in the Euclidean space can be simply computed from the important dual coefficients of the linear combinations by means of solving a simple optimization problem.

Another important idea behind the proposed dual representation is to consider the example-based explanation. It turns out that the dual explanation inherently leads to the example-based explanation when we study how each dual feature λ_*i*_ contributes into predictions. The contribution can be determined by applying well-known surrogate methods, for example, LIME or the neural additive model (NAM) (Agarwal et al., 2021), but the corresponding surrogate models are constructed for features λ but not for initial features.

For the local explanation, we construct the convex hull by using only a part of training data. Though the same algorithm can be successfully applied to the global explanation. In this case, the convex hull covers the entire dataset.

Our contributions can be summarized as follows:

1. A new feature-based explanation method is proposed. It is based on the dual representation of datasets such that generation of new instances in carried out by means of generating points from the uniform distribution in the unit simplex. In other words, the method replaces the perturbation process of feature vectors in the Euclidean space by the uniform generation of points in the unit simplex, which is simpler and is carried out by many well-known algorithms (Rubinstein and Kroese, 2008; Smith and Tromble, 2004). The generation resolves the problem of out-of-domain data and reduces the number of hyperparameters which have to be tuned for perturbing new instances.
2. A new example-based explanation method is proposed. It is again based on the dual representation of datasets and uses well-known explanation models NAM, accumulated local effect (Apley and Zhu, 2020), the linear regression model. The explanation method provides shape function which describe contributions of the dual features into the predictions. In sum, the model chooses the most influential instances among a certain number of nearest neighbors for the explained instance.
3. The proposed methods are illustrated by means of numerical experiments with synthetic and real data. The code of the proposed algorithm can be found in https://github.com/Kozlov992/Dual-Explanation.

The study is organized as follows. Related work can be found in Section 2. A brief introduction to the convex hull, the explanation methods LIME, SHAP, NAM, and example-based methods is given in Section 3. A detailed description of the proposed approach applied to the feature-based explanation and the example-based explanation is available in Section 4. Numerical experiments with synthetic data and real data studying the feature-based explanation are given in Section 5. Section 6 provides numerical examples illustrating example-based explanation. Advantages and limitations of the proposed methods are discussed in Section 7. Concluding remarks can be found in Section 8.

## 2 Related work

### 2.1 Local and global explanation methods

The requirement of the black-box model explanation led to development of many explanation methods. A large part of methods follows from the original LIME method (Ribeiro et al., 2016). These methods include ALIME (Shankaranarayana and Runje, 2019), Anchor LIME (Ribeiro et al., 2018), LIME-Aleph (Rabold et al., 2020), SurvLIME (Kovalev et al., 2020), LIME for tabular data (Garreau and von Luxburg, 2020a,b), GraphLIME (Huang et al., 2022), etc.

To generalize the simple linear explanation surrogate model, several neural network models, including NAM (Agarwal et al., 2021), GAMI-Net (Yang et al., 2021), and AxNNs (Chen et al., 2020), were proposed. These models are based on applying the GAM (Hastie and Tibshirani, 1990). Similar explanation models, including Explainable Boosting Machine (Nori et al., 2019) and EGBM (Konstantinov and Utkin, 2021), were developed using the gradient boosting machine.

Another large part of explanation methods is based on the original SHAP method (Strumbelj and Kononenko, 2010) which uses Shapley values (Lundberg and Lee, 2017) as measures of the feature contribution into the black-box model prediction. This part includes FastSHAP (Jethani et al., 2022), Kernel SHAP (Lundberg and Lee, 2017), Neighborhood SHAP (Ghalebikesabi et al., 2021), SHAFF (Benard et al., 2022), TimeSHAP (Bento et al., 2021), X-SHAP (Bouneder et al., 2020), ShapNets (Wang et al., 2021), etc.

Many explanation methods, including LIME and its modifications, are based on perturbation techniques (Fong and Vedaldi, 2019, 2017; Petsiuk et al., 2018; Vu et al., 2019), which stem from the well-known property that contribution of a feature can be determined by measuring how a prediction changes when the feature is altered (Du et al., 2019). The main difficulty of using the perturbation technique is its computational complexity when samples are of the high dimensionality.

Another interesting group of explanation methods, called the example-based explanation methods (Molnar, 2019), is based on selecting influential instances from a training set having the largest impact on the predictions and its comparison with the explainable instance. Several approaches to the example-based method implementation were considered in Adhikari et al. (2019), Cai et al. (2019), Chong et al. (2022), Crabbe et al. (2021), and Teso et al. (2021).

In addition to the aforementioned methods, there are a huge number of other approaches to solving the explanation problem, for example, Integrated Gradients (Sundararajan et al., 2017), and Contrastive Examples (Dhurandhar et al., 2018). Detailed surveys of many methods can be found in Adadi and Berrada (2018), Arrieta et al. (2020), Bodria et al. (2023), Burkart and Huber (2021), Carvalho et al. (2019), Islam et al. (2022), Guidotti et al. (2019), Li et al. (2022), Rudin (2019), and Rudin et al. (2021).

### 2.2 Convex hull methods and the convex duality concept

Most papers considering the convex hull methods study the relationship between location of decision boundaries and convex hulls of a training set. The corresponding methods are presented in Chau et al. (2013), El Mrabti et al. (2024), Gu et al. (2020), Nemirko and Dula (2021a), Nemirko and Dula (2021b), Renwang et al. (2022), Rossignol et al. (2024), Singh and Kumar (2021), Wang et al. (2013), Yousefzadeh (2020), and Zhang X. et al. (2021). Boundary of the dataset's convex hull is studied in Balestriero et al. (2021) to discriminate interpolation and extrapolation occurring for a sample. Efficient algorithms for efficient computation of the convex hull for training data are presented in Khosravani et al. (2016).

The concept of duality was also widely used in machine learning models starting from duality in the support vector machine and its various modifications (Bennett and Bredensteiner, 2000; Zhang, 2002). This concept was successfully applied to some types of neural networks (Ergen and Pilanci, 2020, 2021), including GANs (Farnia and Tse, 2018), to models dealing with the high-dimensional data (Yao et al., 2018).

At the same time, the aforementioned approaches did not apply to explanation models. Concepts of the convex hull and the convex duality may be a way to simplify and to improve the explanation models.

## 3 Preliminaries

### 3.1 Convex hull

According to Rockafellar (1970), a domain produced by a set of instances as vectors in Euclidean space is convex if a straight line segment that joins every pair of instances belonging to the set contains a vector belonging to the domain. A set S is convex if, for every pair, u,v∈S, and all λ ∈ [0, 1], the vector (1 − λ)**u** + λ**v** belongs to S.

Moreover, if S is a convex set, then for any **x**_1_, **x**_2_, ..., **x**_*t*_ belonging to S and for any non-negative numbers λ_1_, ..., λ_*t*_ such that λ_1_ +... + λ_*t*_ = 1, the sum λ_1_**x**_1_ + ... + λ_*t*_**x**_*t*_ is called a convex combination of **x**_1_, ..., **x**_*t*_. The *convex hull* or *convex envelope* of set X of instances in the Euclidean space can be defined in terms of convex sets or convex combinations as the minimal convex set containing X, or the intersection of all convex sets containing X, or the set of all convex combinations of instances in X.

### 3.2 LIME, SHAP, NAM, and example-based methods

Let us briefly introduce the most popular explanation methods.

*LIME* (Ribeiro et al., 2016) proposes to approximate a black-box explainable model, denoted as *f*, with a simple function *g* in the vicinity of the point of interest **x**, whose prediction by means of *f* has to be explained, under condition that the approximation function *g* belongs to a set of explanation models *G*, for example, linear models. To construct the function *g*, a new dataset consisting of generated points around **x** is constructed with predictions computed be means of the black-box model. Weights *w*_**x**_ are assigned to new instances in accordance with their proximity to point **x** by using a distance metric, for example, the Euclidean distance. The explanation function *g* is obtained by solving the following optimization problem:


(1) $$\begin{array}{l} \arg\underset{g\in G}{\min}L\left(f,g,w_{\text{x}}\right)+\text{Φ}\left(g\right). \end{array}$$

Here, *L* is a loss function, for example, mean squared error, which measures how the function *g* is close to function *f* at point **x**; Φ(*g*) is the model complexity. A local linear model is the result of the original LIME such that its coefficients explain the prediction.

Another approach to explaining the black-box model predictions is *SHAP* (Lundberg and Lee, 2017; Strumbelj and Kononenko, 2010), which is based on a concept of the Shapley values (Shapley, 1953) estimating contributions of features to the prediction. If we explain prediction *f*(**x**_0_) from the model at a local point **x**_0_, then the *i*-th feature contribution is defined by the Shapley value as


(2) $$\begin{array}{l} \phi_{i}=\underset{S\subseteq N\backslash \left\{i\right\}}{\sum }\frac{|S|!\left(|N|-|S|-1\right)!}{|N|!}\left[f\left(S\cup \left\{i\right\}\right)-f\left(S\right)\right], \end{array}$$

where *f*(*S*) is the black-box model prediction under condition that a subset *S* of the instance **x**_0_ features is used as the corresponding input; *N* is the set of all features.

It can be seen from Equation 2 that the Shapley value ϕ_*i*_ can be regarded as the average contribution of the *i*-th feature across all possible permutations of the feature set. The prediction *f*(**x**_0_) can be represented by using Shapley values as follows (Lundberg and Lee, 2017; Strumbelj and Kononenko, 2010):


(3) $$\begin{array}{l} f\left({\text{x}}_{0}\right)=\text{𝔼}\left[f\left(\text{x}\right)\right]+\overset{m}{\underset{j=1}{\sum }}\phi_{j}. \end{array}$$

To generalize LIME, *NAM* was proposed in Agarwal et al. (2021). It is based on the generalized additive model of the form *y*(**x**) = *g*_1_(*x*_1_) + ... + *g*_*m*_(*x*_*m*_) (Hastie and Tibshirani, 1990) and consists of *m* neural networks such that a single feature is fed to each subnetwork and each network implements function *g*_*i*_(*x*_*i*_), where *g*_*i*_ is a univariate shape function with *E*(*g*_*i*_) = 0. All networks are trained jointly using backpropagation and can learn arbitrarily complex shape functions (Agarwal et al., 2021). The loss function for training the whole neural network is of the form:


(4) $$\begin{array}{l} L=\overset{n}{\underset{i=1}{\sum }}{\left(y_{i}-\overset{m}{\underset{k=1}{\sum }}g_{k}\left(x_{k}^{\left(i\right)}\right)\right)}^{2}, \end{array}$$

where $x_{k}^{\left(i\right)}$ is the *k*-th feature of the *i*-th instance; *n* is the number of training instances.

The representation of results in NAM in the form of shape functions can be considered in two ways. On the one hand, the functions are more informative, and they show how features contribute into a prediction. On the other hand, we often need to have a single value of the feature contribution which can be obtained by computing an importance measure from the obtained shape function.

NAM significantly extends the flexibility of explanation models due to possibility to implement arbitrary functions of features by means of neural networks.

According to Molnar (2019), an instance or a set of instances are selected in *example-based explanation methods* to explain the model prediction. In contrast to the feature importance explanation (LIME, SHAP), the example-based methods explain a model by selecting instances from the dataset and do not consider features or their importance for explaining. In the context of obtained results, the example-based methods are represented by influential instances (points from the training set that have the largest impact on the predictions) and by prototypes (representative instances from the training data). It should be noted that instances used for explanation may not belong to a dataset and are combinations of instances from the dataset or some points in the dataset domain. The well-known method of *K* nearest neighbors can be regarded as an example-based explanation method.

## 4 Materials and methods

### 4.1 Dual explanation

Let us consider the method for dual explanation. Suppose that there is a dataset $T=\left\{\left({\text{x}}_{1},y_{1}\right),...,\left({\text{x}}_{t},y_{t}\right)\right\}$ of *t* points (**x**_*i*_, *y*_*i*_), where ${\text{x}}_{i}=\left(x_{1}^{\left(i\right)},...,x_{m}^{\left(i\right)}\right)\in X\subset {\mathbb{R}}^{m}$ is a feature vector consisting of *m* features, *y*_*i*_ is the observed output for the feature vector **x**_*i*_ such that *y*_*i*_ ∈ ℝ in the regression problem and *y*_*i*_ ∈ {1, 2, ..., *C*} in the classification problem with *C* classes. It is assumed that output *y* of an explained black-box model is a function *f*(**x**) of an associated input vector **x** from X.

To explain an instance ${\text{x}}_{0}\in X$, an interpretable surrogate model *g* for the black-box model *f* is trained in a local region around **x**_0_. It is carried out by generating a new dataset S of *n* perturbed samples in the vicinity of the point of interest **x**_0_ similarly to LIME. Samples are assigned by weights *w*_**x**_ in accordance with their proximity to the point **x**. By using the black-box model, output values *y* are obtained as function *f* of generated instances. As a result, dataset S consists of *n* pairs (**x**_*i*_, *f*(**x**_*i*_)), *i* = 1, ..., *n*. Interpretable surrogate model *g* is now trained on S. Many explanation methods such as LIME and SHAP are based on applying the linear regression function


(5) $$\begin{array}{l} g\left(\text{x}\right)=a_{1}x_{1}+...+a_{m}x_{m}={\text{ax}}^{\text{T}}, \end{array}$$

as an interpretable model because each coefficient *a*_*i*_ in *g* quantifies how the *i*-th feature impacts on the prediction. Here **a** = (*a*_1_, ..., *a*_*m*_). It should be noted that the domain of set S coincides with the domain of set T in the case of the global explanation.

Let us consider the convex hull P of a set of *K* nearest neighbors of instance **x**_0_ in the Euclidean space. The convex hull P forms a convex polytope with *d* vertices or extreme points ${\text{x}}_{i}^{*}$, *i* = 1, ..., *d*. Then, each point x∈P is a convex combination of *d* extreme points:


(6) $$\begin{array}{l} \text{x}=\overset{d}{\underset{i=1}{\sum }}{\text{λ}}_{i}{\text{x}}_{i}^{*},\;\text{where }{\text{λ}}_{i}\geq 0,\;\overset{d}{\underset{i=1}{\sum }}{\text{λ}}_{i}=1. \end{array}$$

This implies that we can uniformly generate a vector in the unit simplex of possible vectors λ consisting of *d* coefficients λ_1_, ..., λ_*d*_, denoted Δ^*d*−1^. In other words, we can consider points in the unit simplex Δ^*d*−1^ and construct a new dual dataset $D=\left\{\left({\text{λ}}^{\left(1\right)},z_{1}\right),...,\left({\text{λ}}^{\left(n\right)},z_{n}\right)\right\}$, which consists of vectors ${\text{λ}}^{\left(j\right)}=\left({\text{λ}}_{1}^{\left(j\right)},...,{\text{λ}}_{d}^{\left(j\right)}\right)$, and the corresponding values *z*_*j*_, *j* = 1, ..., *n*, computed by using the black-box model *f* as follows:


(7) $$\begin{array}{l} z_{j}=f\left(\overset{d}{\underset{i=1}{\sum }}{\text{λ}}_{i}^{\left(j\right)}{\text{x}}_{i}^{*}\right), \end{array}$$

i.e., *z*_*j*_ is a prediction of the black-box model when its input is vector $\overset{d}{\underset{i=1}{\sum }}{\text{λ}}_{i}^{\left(j\right)}{\text{x}}_{i}^{*}.$

In sum, we can train the “dual” linear regression model (the surrogate model) for explanation on dataset D, which is of the form:


(8) $$\begin{array}{l} h\left(\text{λ}\right)=b_{1}{\text{λ}}_{1}+...+b_{d}{\text{λ}}_{d}=\text{b}{\text{λ}}^{\text{T}}, \end{array}$$

where **b** = (*b*_1_,..., *b*_*d*_) is the vector of coefficients of the “dual” linear regression model.

The surrogate model can be trained by means of LIME or SHAP with the dual dataset D.

Suppose that we have trained the function *h*(λ) and computed coefficients *b*_1_, ..., *b*_*d*_. The next question is how to transform these coefficients to coefficients *a*_1_, ..., *a*_*m*_ which characterize the feature contribution into the prediction. In the case of the linear regression, coefficients of function *g*(**x**) = *a*_1_*x*_1_ + ... + *a*_*m*_*x*_*m*_ can be found from the condition:


(9) $$\begin{array}{l} g\left(\overset{d}{\underset{i=1}{\sum }}{\text{λ}}_{i}^{\left(j\right)}{\text{x}}_{i}^{*}\right)=h\left({\text{λ}}_{j}\right), \end{array}$$

which has to be satisfied for all generated λ_*j*_. This obvious condition means that predictions of the “primal” surrogate model with coefficients *a*_1_, ..., *a*_*m*_ has to coincide with predictions of the “dual” model.

Introduce a matrix consisting of extreme points


(10) $$\begin{array}{l} \text{x}={\left({\text{x}}_{i}^{*\text{T}}\right)}_{i=1}^{d}. \end{array}$$

Note that, λ_*i*_ = 1 and λ_*j*_ = 0, *j* ≠ *i*, for the *i*-th extreme point. This implies that the condition (Equation 9) can be rewritten as


(11) $$\begin{array}{l} g\left({\text{x}}_{i}^{*}\right)=\;h\left(0,...,1_{i},...,0\right)=b_{i}. \end{array}$$

By using Equation 5, we get


(12) $$g\left(x_{i}^{*}\right)=ax_{i}^{*T}=b_{i}.$$

Hence, there holds


(13) $$\begin{array}{l} \text{aX}=\text{b}. \end{array}$$

It follows from the above that


(14) $$\begin{array}{l} {\text{a}=\text{X}}^{-1}\text{b}, \end{array}$$

where **X**^−1^ is the pseudoinverse matrix.

Generally, the vector **a** can be computed by solving the following unconstrained optimization problem:


(15) $$\begin{array}{l} {\text{a}}_{opt}=\arg\underset{\text{a}\in {\mathbb{R}}^{m}}{\text{min}}∥\text{aX}-\text{b}{∥}^{2}. \end{array}$$

In the original LIME, perturbed instances are generated around **x**_0_. One of the important advantages of the proposed dual approach is the opportunity to avoid generating instances in accordance with a probability distribution with parameters and to generate only uniformly distributed points λ^(*j*)^ in the unit simplex Δ^*d*−1^. Indeed, if we have image data, then it is difficult to perturb pixels or superpixels of images. Moreover, it is difficult to determine parameters of the generation to cover instances from different classes. According to the dual representation, after generating vectors λ^(*j*)^, new vectors **x**_*j*_ are computed by using Equation 6. This is similar to the mixup method (Zhang et al., 2018) to some extent that generates new samples by linear interpolation of multiple samples and their labels. However, in contrast to the mixup method, the prediction is obtained as the output of the black-box model (see Equation 7), but not as the convex combination of one-hot label encodings. Another important advantage is that instances corresponding to the generated set D are totally included in the domain of the dataset T. This implies that we do not get anomalous predictions *f*(**x**_*i*_) when generated **x**_*i*_ is far from the domain of the dataset T.

**Figure 2:**
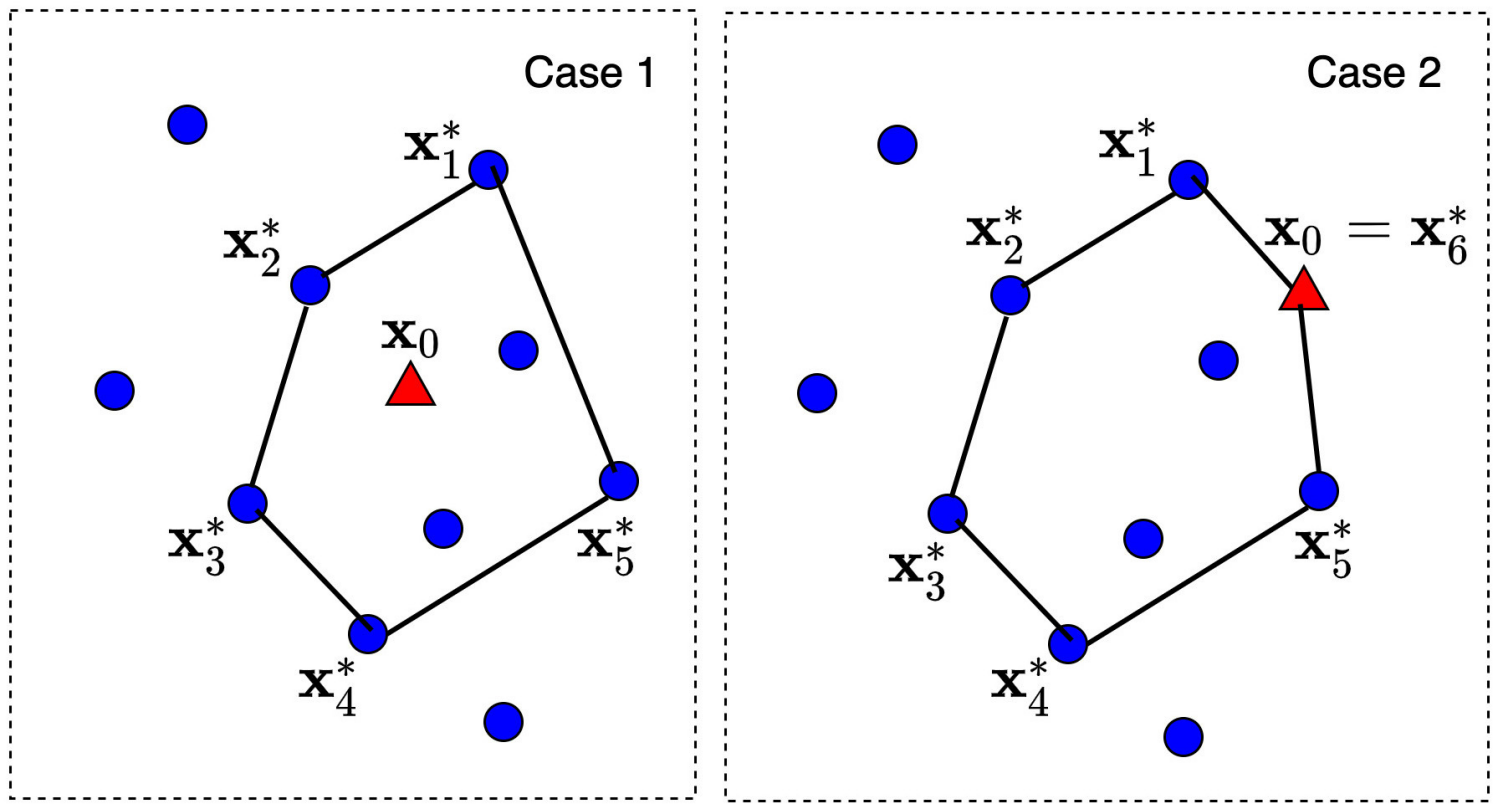
Two cases of the explained point location and the convex polytops constructed from *K* nearest neighbors.

Another question is how to choose the convex hull of the predefined size and, hence, how to determine extreme points ${\text{x}}_{i}^{*}$ of the corresponding convex polytope. The problem is that the convex hull has to include some number of points from dataset T and the explained point **x**_0_. Let us consider *K* nearest neighbors around **x**_0_ from T, where *K* is a tuning parameter satisfying condition *K*≥*d*. The convex hull is constructed on these *K* + 1 points (*K* points from T and one point **x**_0_). Then, there are *d* points among *K* nearest neighbors which define a convex polytope and can be regarded as its extreme points. It should be noted that *d* depends on the dataset analyzed. Figure 2 illustrates two cases of the explained point location and the convex polytopes constructed from *K* = 7 nearest neighbors. The dataset consists of 10 points depicted by circles. A new explained point **x**_0_ is depicted by the red triangle. In Case 1, point **x**_0_ lies in the largest convex polytope with *d* = 5 extreme points ${\text{x}}_{1}^{*},...,{\text{x}}_{5}^{*}$ constructed from seven nearest neighbors. The largest polytope is taken in order to envelop as large as possible points from the dataset. In Case 2, point **x**_0_ lies outside the convex polytope constructed from nearest neighbors. Therefore, this point is included into the set of extreme points and *d* ≤ *K*+1. As a result, we have *d* = 6 extreme points ${\text{x}}_{1}^{*},...,{\text{x}}_{5}^{*},{\text{x}}_{6}^{*}={\text{x}}_{0}$.

To identify whether the newly added point can be expressed as convex combination of the existing vectors, the Farka's lemma (Dinh and Jeyakumar, 2014) can be applied.

Points λ^(*j*)^ from the unit simplex Δ^*d*−1^ are randomly selected in accordance with the uniform distribution over the simplex. This procedure can be carried out by means of generating random numbers in accordance with the Dirichlet distribution (Rubinstein and Kroese, 2008). There are also different approaches to generate points from the unit simplex (Smith and Tromble, 2004).

Finally, we write Algorithm 1 implementing the proposed method.

**Algorithm 1:** The dual explanation algorithm.

**Require:** Training set T; the black-box model *f*; explainable point **x**_0_; the number of nearest neighbors *K*
**Ensure:** Important features of **x**_0_ (vector $\text{a}={\left(a_{1},...,a_{m}\right)}^{\text{T}}$ of the linear surrogate model coefficients)
1: Determine a set $T_{K}$ of *K* nearest neighbors for **x**_0_ adding **x**_0_ itself
2: Construct the largest convex hull P of $T_{K}$
3: Find extreme points of P and their number *d* ≤ *K*+1
4: Generate uniformly *n* points λ^(*j*)^, *j* = 1, ..., *n*, from the unit simplex Δ^*d*−1^
5: Find predictions *z*_*i*_ of the black-box model in accordance with associated input $\overset{d}{\underset{i=1}{\sum }}{\text{λ}}_{i}^{\left(j\right)}{\text{x}}_{i}^{*}$ for all *i* = 1, ..., *n*
6: Construct a new dual dataset $D=\left\{\left({\text{λ}}^{\left(1\right)},z_{1}\right),...,\left({\text{λ}}^{\left(n\right)},z_{n}\right)\right\}$
7: Train the linear regression (Equation 8) on dataset D and find the vector of coefficients $\text{b}={\left(b_{1},...,b_{d}\right)}^{\text{T}}$
8: Find vector **a** by solving optimization problem (Equation 15)

**Figure 3:**
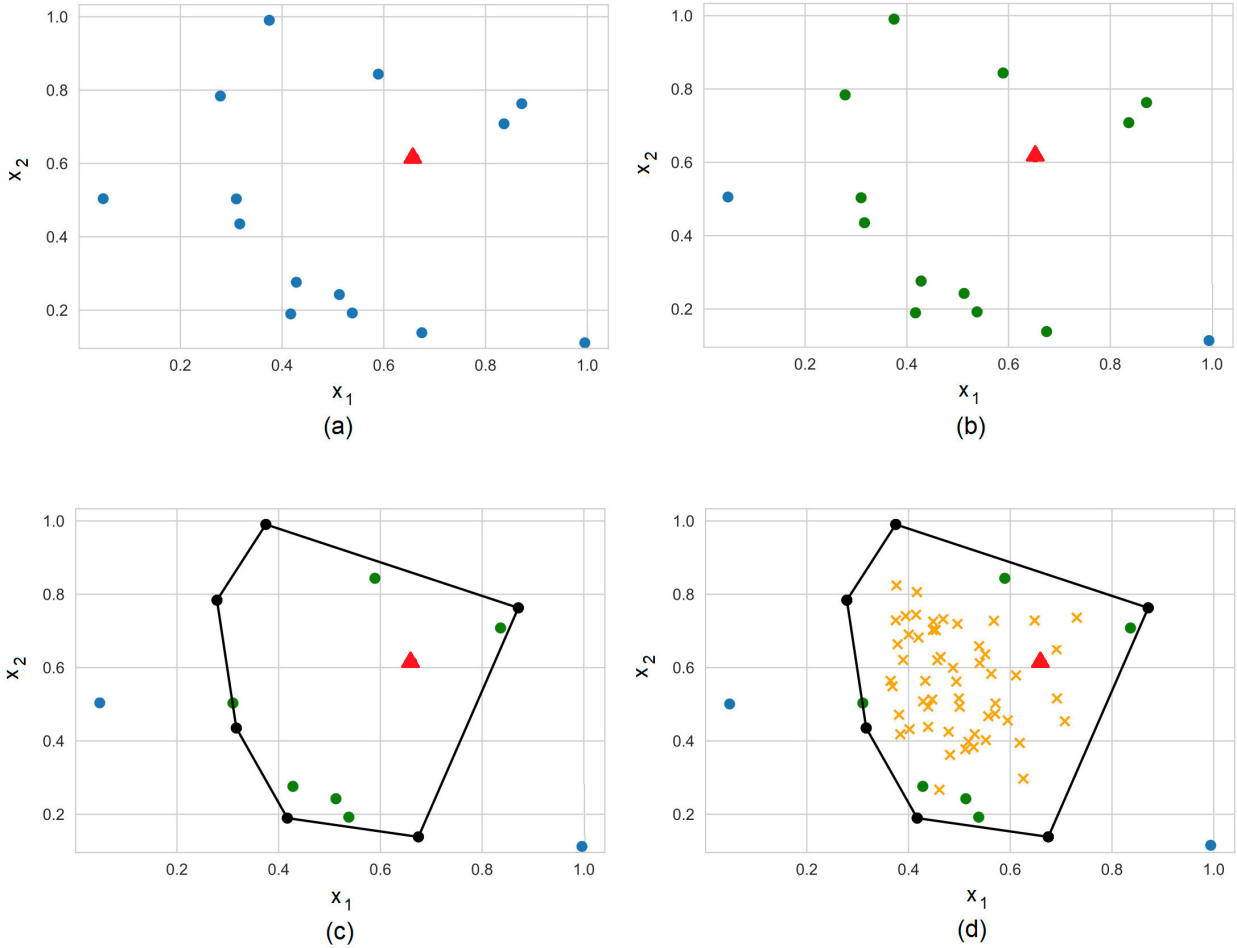
Steps of the algorithm for explanation of a prediction provided by a black-box model at the point depicted by the small triangle. **(A)** Training dataset (circles) and the explainable example (triangle). **(B)** 13 nearest neighbors. **(C)** The convex hull around the explainable example. **(D)** Generated examples (crosses) in the convex hull.

Figure 3 illustrates steps of the algorithm for explanation of a prediction provided by a black-box model at the point depicted by the small triangle. Points of the dataset are depicted by small circles. The training dataset T and the explained point are shown in Figure 3A. Figure 3B shows set $T_{K}$ of *K* = 13 nearest points such that only two points (0.05, 0.5) and (1.0, 0.1) from training set T do not belong to set $T_{K}$. The convex hull and the corresponding extreme points are shown in Figure 3C. Points uniformly generated in the unit simplex are depicted by means of small crosses in Figure 3D. It is interesting to point out that the generated points are uniformly distributed in the unit simplex, but not in the convex polytope as it is follows from Figure 3D. We uniformly generate vectors λ, but the corresponding vectors **x** are not uniformly distributed in the polytope. One can see from Figure 3D that generated points in the initial (primal) feature space tend to be located in the area where the density of extreme points is largest. This is a very interesting property of the dual representation. It means that the method takes into account the concentration of training points and the probability distribution of the instances in the dataset.

**Figure 4:**
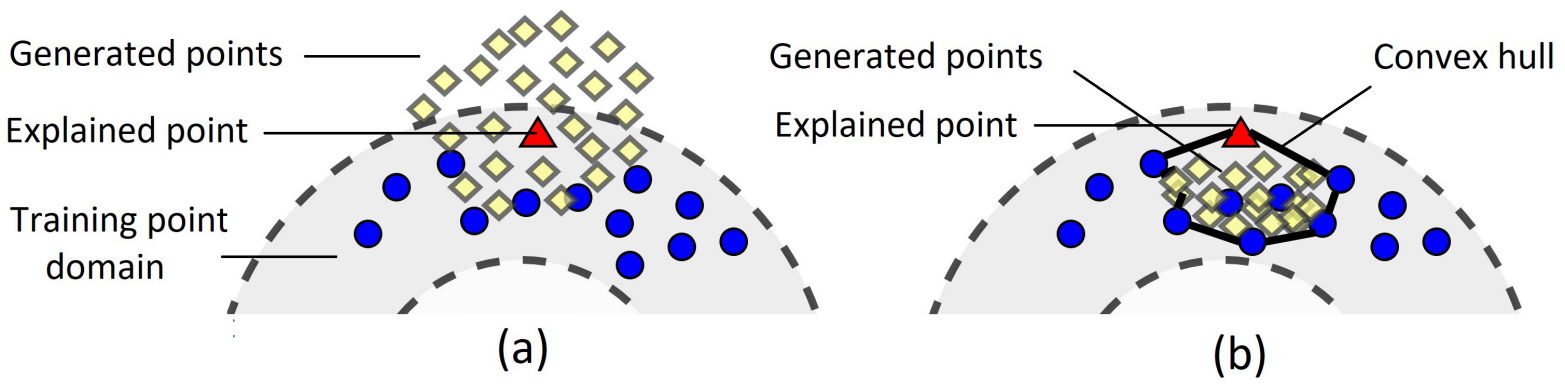
Generated points in the original LIME **(A)** and in the proposed dual method **(B)**.

The difference between points generated by means of the original LIME and the proposed method is illustrated in Figure 4 where the left picture (Figure 4A) shows a fragment of Figure 1 and the right picture (Figure 4B) illustrates how the proposed method generates instances.

The proposed method requires finding all the extreme points (vertices) of the convex hull of a given point ${\text{x}}_{0}\in {\mathbb{R}}^{d}$ and its nearest neighbors **x**_1_, **x**_2_, …, **x**_*n*−1_. When the dimension *d* is small, these extreme points can be computed in time *O*(2^*O*(*d*log*d*)^*n*^2^) = *O*(*n*^2^) (Ottmann et al., 2001). In general, determining whether **x**_*i*_ is an extreme point can be done by checking the condition


(16) $$\begin{array}{l} conv\left({\left\{{\text{x}}_{j}\right\}}_{j=0}^{n-1}\right)\neq conv\left({\left\{{\text{x}}_{j}\right\}}_{j=0}^{n-1}\backslash \left\{{\text{x}}_{i}\right\}\right), \end{array}$$

where *conv*(*P*) denotes the convex hull of the set *P*.

The above condition is equivalent to solving a feasibility problem that can be formulated as a linear program. This linear program involves *n* variables and *n*+*d* constraints and can be solved using the interior-point method described in Vaidya (1989). For each point, the time complexity of this procedure is *O*((*n*+*d*)^3/2^*n*log(*n*)), resulting in an overall complexity of


(17) $$\begin{array}{l} O\left({\left(n+d\right)}^{3/2}n^{2}\log\left(n\right)\right). \end{array}$$

In the extreme case, when *d*≫1, we can use the AVTA algorithm ${\left\{x_{j}\right\}}_{j=0}^{n-1}.$. *This algorithm has the time complexity*O(n^2^(t^−^)), *where*t ∈ (0, 1). The approximation becomes more precise as *t* → 0.

The dual approach can work best when applied to analysis of potential outliers. In that regard, the generation procedure proposed in the study is more robust than the one used in LIME. By choosing the generation region as the convex hull of the explained point nearest neighbors, we reduce the likelihood of creating additional samples that fail to align with the original data distribution. As for hyperparameters, the number of nearest neighbors used to construct the convex hull for the explained point largely depends on the user's preferences and the nature of analyzed data. We can stop incorporating additional neighbors when a certain threshold is reached, such as when the next nearest neighbor is considerably more distant compared to the previous ones. Furthermore, we can choose to exclude a new neighbor if its data features clearly indicate that it would not contribute much to the analysis of the explained point. The number of points to generate can be taken as *k*·*n*, where *k* is a real number and *n* is the number of selected neighbors. By default, *k* = 3. This implies that we can increment the number of generated points until we observe the convergence of dual coefficients. We can also modify the distribution type employed for creating the dual dataset. For instance, if we take new points to be generated mostly in close proximity to the explained point **x** = (*x*_1_, ..., *x*_*d*_), we can sample the points from the Dirichlet distribution with concentration parameters α_*i*_ = 1 + *t* · *x*_*i*_, where *t* > 0.

### 4.2 Example-based explanation and NAM

It turns out that the proposed method for the dual explanation inherently leads to the example-based explanation. An example-based explainer justifies the prediction on the explainable instance by returning instances related to it. Let us consider the dual representation (Equation 8). If we normalize coefficients **b** = (*b*_1_, ..., *b*_*d*_) as


(18) $$v_{i}=\frac{b_{i}}{\sum_{j=1}^{d}b_{j}},$$

then new coefficients (*v*_1_, ..., *v*_*d*_) quantify how extreme points $\left({\text{x}}_{1}^{*},...,{\text{x}}_{d}^{*}\right)$ associated with (λ_1_, ..., λ_*d*_) impact on the prediction. The greater the value of *v*_*i*_, the greater contribution of ${\text{x}}_{i}^{*}$ into a prediction. Hence, the linear combination of extreme points


(19) $$\begin{array}{l} \text{x}=\overset{d}{\underset{i=1}{\sum }}v_{i}{\text{x}}_{i}^{*} \end{array}$$

allows us to get an instance **x** explaining **x**_0_.

An outstanding approach considering convex combinations of instances from a dataset as the example-based explanation was proposed in Crabbe et al. (2021). In fact, we came to the similar example-based explanation by using the dual representation and constructing linear regression surrogate model for new variables (λ_1_, ..., λ_*d*_).

**Figure 5:**
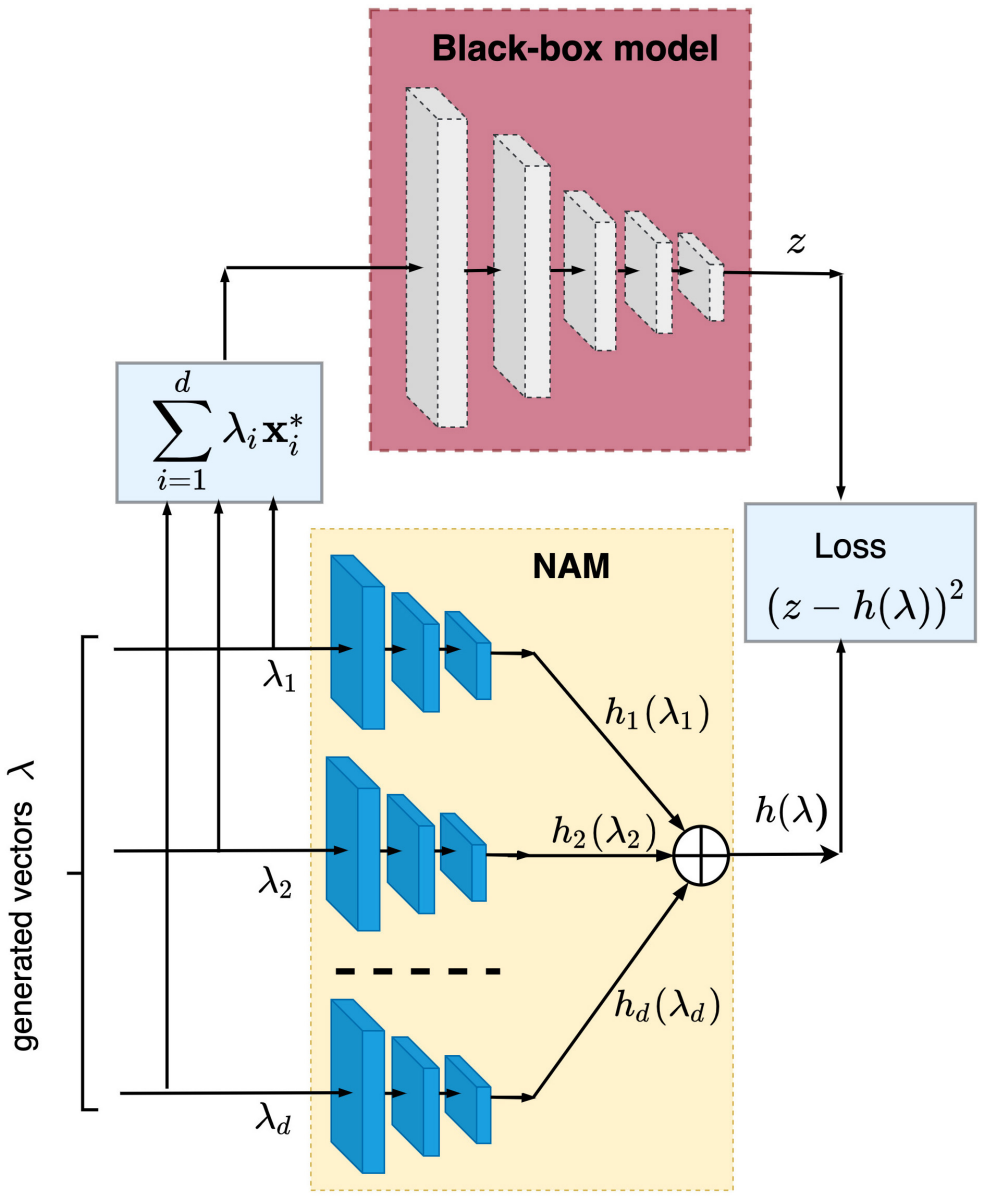
Scheme of training NAM on the generated set of random vectors λ.

The example-based explanation may be very useful when we apply NAM (Agarwal et al., 2021) for explaining the black-box prediction. By using dual dataset $D=\left\{\left({\text{λ}}^{\left(1\right)},z_{1}\right),...,\left({\text{λ}}^{\left(n\right)},z_{n}\right)\right\}$, we train NAM consisting of *d* subnetworks such that each subnetwork implements the shape function *h*_*i*_(λ_*i*_). Figure 5 illustrates a scheme of training NAM. Each generated vector λ is fed to NAM such that each its variable λ_*i*_ is fed to a separate neural subnetwork. For the same vector λ, the corresponding instance **x** is computed by using Equation 6, and it is fed to the black-box model. The loss function for training the whole neural network is defined as the difference between the output *z* of the black-box model and the sum of shape functions *h*_1_, ..., *h*_*d*_ implemented by neural subnetworks for the corresponding vector λ, i.e., the loss function *L* is of the form:


(20) $$\begin{array}{l} L=\overset{n}{\underset{i=1}{\sum }}{\left(z_{i}-\overset{d}{\underset{k=1}{\sum }}h_{k}\left({\text{λ}}_{k}^{\left(i\right)}\right)\right)}^{2}+\alpha R\left(\text{w}\right), \end{array}$$

where ${\text{λ}}_{k}^{\left(i\right)}$ is the *k*-th element of vector **λ**^(*i*)^; *R* is a regularization term with the hyperparameter α which controls the strength of the regularization; **w** is the vector of the neural network training parameters.

The main difficulty of using the NAM results, i.e., shape functions *h*_*k*_(λ_*k*_), is how to interpret the shape functions for explanation. However, in the context of the example-based explanation, this difficulty can be simply resolved. First, we study how a shape function can be represented by a single value characterizing the importance of each variable λ_*k*_, *k* = 1, ..., *d*. The shape function is similar to the partial dependence plot (Friedman, 2001; Molnar, 2019) to some extent. The importance of a variable (λ_*k*_) can be evaluated by studying how rapidly the shape function, corresponding to the variable, is changed. The rapid change of the shape function says that small changes of the variable significantly change the target values (*z*). The above implies that we can use the importance measure proposed in Greenwell et al. (2018), which is defined as the deviation of each unique variable value from the average curve. In terms of the dual variables, it can be written as:


(21) $$\begin{array}{l} I\left({\text{λ}}_{k}\right)=\sqrt{\frac{1}{r-1}\overset{r}{\underset{i=1}{\sum }}{\left(h_{k}\left({\text{λ}}_{k}^{\left(i\right)}\right)-\frac{1}{r}\overset{r}{\underset{i=1}{\sum }}h_{k}\left({\text{λ}}_{k}^{\left(i\right)}\right)\right)}^{2}}, \end{array}$$

where *r* is a number of values of each variable λ_*k*_, which are analyzed to study the corresponding shape function.

Normalized values of the importance measures can be regarded as coefficients *v*_*i*_, *i* = 1, ..., *d*, in Equation 19, i.e., they show how important each extreme point or how each extreme point can be regarded as an instance which explains instance **x**_0_.

An additional important advantage of the dual representation is that shape functions for all variables λ_*k*_, *k* = 1, ..., *d*, have the same scale because all variables are in the interval from 0 to 1. This allows us to compare the importance measures *I*(λ_*k*_) without the preliminary scaling which can make results incorrect.

## 5 Numerical experiments with the feature-based explanation

### 5.1 Example 1

First, we consider the following simplest example when the black-box model is of the form:


$$\begin{array}{l} f\left(\text{x}\right)=10x_{1}-20x_{2}-2x_{3}+3x_{4}+0x_{5}+0x_{6}+0x_{7}+\xi \\ \;=\text{ax}+\xi ,\;\xi \sim N\left(0,0.1\right). \end{array}$$

Let us estimate the feature importance by using the proposed dual model. We generate *n* = 1000 points **x**_*i*_, *i* = 1, ..., *N*, with components uniformly distributed in interval [0, 1], which are explained. For every point **x**_*i*_, the dual model with *K* = 10 nearest neighbors is constructed by generating 30 vectors λ^(*i*)^ ∈ ℝ^7^ in the unit simplex. By applying Algorithm 1, we compute optimal vector ${\text{a}}^{\left(i\right)}={\left(a_{1},...,a_{7}\right)}^{\text{T}}$ for every point **x**_*i*_. We expect that the mean value $\bar{\text{a}}$ of **a**^(*i*)^ over all *i* = 1, ..., *N* should be as close as possible to the true vector of coefficients **a** forming function *f*(**x**). The corresponding results are shown in Table 1. It can be seen from Table 1 that the obtained vector $\bar{\text{a}}$ is actually close to vector **a**.

**Table 1:** Values of the importance measures in Example 1 in accordance with the explanation approach LR.

	***x*_1_**	***x*_2_**	***x*_3_**	***x*_4_**	***x*_5_**	***x*_6_**	***x*_7_**
**a**	10	−20	−2	3	0	0	0
$\bar{\text{a}}$	9.98	−20.01	−2.02	2.97	0.11	−0.02	0.03

### 5.2 Example 2

Let us consider another numerical example where the non-linear black-box model is investigated. It is of the form:


$$f\left(\text{x}\right)=-x_{1}^{2}+2x_{2}+\xi ,\;\xi \sim N\left(0,0.05\right).$$

We take *N* = 400 and generate two sets of points **x**. The first set contains **x** whose features are uniformly generated in the interval [0, 1]. The second set consists of **x** whose features are uniformly generated in the interval [15, 16]. It is interesting to note that the feature *x*_1_ is more important for the case of the second set because $x_{1}^{2}$ rapidly increases whereas $x_{1}^{2}$ decreases when we consider the first set and *x*_2_ is more important in this case.

We take *K* = 6 and generate 30 vectors λ^(*i*)^ uniformly distributed in the unit simplex for every **x** to construct the linear model *h*(λ^(*i*)^). Mean values of the normalized importance of features *x*_1_ and *x*_2_ obtained for the first set are −0.3 and 0.86 and for the second set are −0.95 and 0.37. These results completely coincide with the importance of features considered above for two subsets.

### 5.3 Example 3

A goal of the following numerical example is to consider a case when we try to get predictions for points lying outside bounds of data on which the black-box model was trained as it is depicted in Figure 1. In this case, the predictions of generated instances may be inaccurate and can seriously affect quality of many explanation models, for example, LIME, which uses the perturbation technique.

The initial dataset consists of *n* = 400 feature vectors **x**_1_, ..., **x**_*n*_ such that there holds


(22) $$\begin{array}{l} {\text{x}}_{i}=\left(\begin{array}{c} x_{i}^{\left(1\right)}\\ x_{i}^{\left(2\right)} \end{array}\right)=\rho \left(\begin{array}{c} \cos\varphi \\ \sin\varphi \end{array}\right), \end{array}$$

where parameter ρ^2^ is uniformly distributed in interval [0, 2^2^]; parameter φ is uniformly distributed in interval [0, 2π].

The observed outputs *y*_*i*_ = *f*(**x**_*i*_) are defined as


(23) $$\begin{array}{l} f\left({\text{x}}_{i}\right)={\left(x_{i}^{\left(1\right)}\right)}^{2}+{\left(x_{i}^{\left(2\right)}\right)}^{2}+\xi ,\;\xi \sim N\left(0,0.05\right). \end{array}$$

We use two black-box models: the KNN regressor with *k* = 6 and the random forest consisting of 100 decision trees, implemented by means of the Python Sckit-learn. The above black-box models have default parameters taken from Sckit-learn.

**Figure 6:**
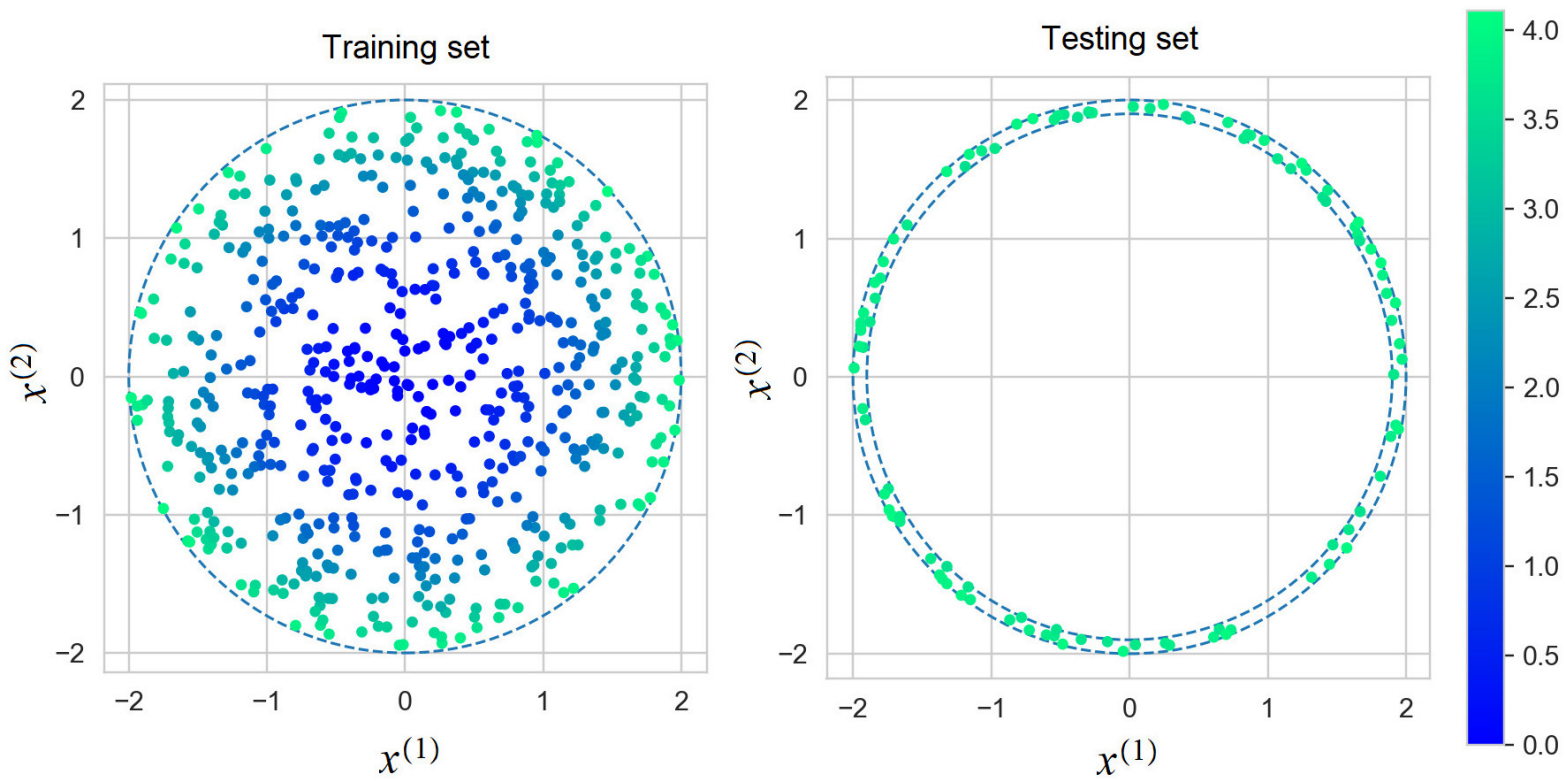
Instances for training the black-box models **(the left picture)** and testing points for evaluation of the explanation models **(the right picture)**.

We construct the explanation models at *l* = 100 testing points **x**_1, *test*_, ..., **x**_*l, test*_ of the form Equation 22, but with parameters ρ^2^ uniformly distributed in [1.9^2^, 2^2^] and φ uniformly distributed in [0, 2π]. It can be seen from the interval of parameter ρ that a part of generated points can be outside bounds of training data **x**_1_, ..., **x**_*n*_. Figure 6 shows the set of instances for training the black-box model and the set of testing instances for evaluation of the explanation models.

The dual model is constructed in accordance with Algorithm 1 using *K* = 6 nearest neighbors. We generate 30 dual vectors λ^(*j*)^ to train the dual model. We also use LIME and generate 30 points having normal distribution $N\left({\text{x}}_{j,test},\Sigma \right)$, where Σ = diag(0.05, 0.05). Every point has a weight generated from the normal distribution with parameter *v* = 0.01.

To compare the dual model and LIME, we use the mean squared error (MSE) which measures how predictions of the explanation model *g*(**x**) are close to predictions of the black-box model *f*(**x**) (KNN or the random forest). It is defined as


$$MSE=\frac{1}{l}\overset{l}{\underset{j=1}{\sum }}{\left(f\left({\text{x}}_{j,test}\right)-g\left({\text{x}}_{j,test}\right)\right)}^{2}.$$

Values of the MSE measures for the dual explanation model and for the original LIME, when KNN is used as a black-box model, are 0.01 and 0.02, respectively. It can be seen from the results that the dual model provides better results in comparison with LIME because some generated points in LIME are located outside the training domain. Values of the MSE measures for the dual explanation model and for the original LIME, when the random forest is used as a black-box model, are 0.005 and 0.014, respectively.

### 5.4 Example 4

Let us perform a similar experiment with real data by taking the dataset “Combined Cycle Power Plant Data Set” (https://archive.ics.uci.edu/ml/datasets/combined+cycle+power+plant) consisting of 9568 instances having 4 features. We use Z-score normalization (the mean is 0 and the standard deviation is 1) for feature vectors from the dataset. Two black-box models implemented by using the KNN regressor with *K* = 10 and the random forest regressor consisting of 100 decision trees. The testing set consisting of *l* = 200 new instances is produced as follows. The convex hull of the training set in the 4-dimensional feature space is determined. Then, vertices of the obtained polytope are computed. Two adjacent vertices **x**_*j*_1__ and **x**_*j*_2__ are randomly selected. Value λ is generated from the uniform distribution on the unit interval. A new testing instance **x**_*j, test*_ is obtained as **x**_*j, test*_ = λ**x**_*j*_1__+(1−λ)**x**_*j*_2__. Then, we again select adjacent vertices and repeat the procedure for computing testing instances *l* times. As a result, we get the testing set **x**_*j, test*_, *j* = 1, ..., *l*.

The dual model is constructed in accordance with Algorithm 1 using *K* = 10 nearest neighbors. We again generate 30 dual vectors λ^(*j*)^ to train the dual model. We also use LIME and generate 30 points having normal distribution $N\left({\text{x}}_{j,test},\Sigma \right)$, where Σ = diag(0.05, 0.05, 0.05, 0.05). Every point has a weight generated from the normal distribution with parameter *v* = 0.5.

Values of the MSE measures for the dual explanation model and for the original LIME, when KNN is used as a black-box model trained on dataset “Combined Cycle Power Plant Data Set”, are 84 and 0173, respectively. It can be seen from the results that the dual model provides better results in comparison with LIME because some generated points in LIME are located outside the training domain. Values of the MSE measures for the dual explanation model and for the original LIME, when the random forest is used as a black-box model, are 110 and 282, respectively. One can again see from the above results that the dual models outperform LIME.

## 6 Numerical experiments with the example-based explanation

### 6.1 Example 1

We start from the synthetic instances illustrating the dual example-based explanation when NAM is used. Suppose that the explained instance **x**_0_ belongs to a polytope with six vertices **x**_1_, ..., **x**_6_ (*d* = 6). The black-box model is a function *f*(**x**) such that


(24) $$\begin{array}{l} f\left(\text{x}\right)=f\left(\overset{6}{\underset{k=1}{\sum }}{\text{λ}}_{k}{\text{x}}_{k}\right)=h\left(\text{λ}\right)=h\left({\text{λ}}_{1},...,{\text{λ}}_{6}\right)\\ \;=15{\text{λ}}_{1}+22{\text{λ}}_{2}+0{\text{λ}}_{3}+40\left(1-{\text{λ}}_{4}\right)\sin\left(3.14\cdot {\text{λ}}_{4}\right)+0{\text{λ}}_{5}+0{\text{λ}}_{6}. \end{array}$$

*n* = 2, 000 vectors λ^(*i*)^ ∈ ℝ^6^, *i* = 1, ..., *n*, are uniformly generated in the unit simplex Δ^6 − 1^. For each point λ^(*i*)^, the corresponding prediction *z*_*i*_ is computed by using the black-box function *h*(λ). NAM is trained with the learning rate 0.0005, with hyperparameter α = 10^−4^, the number of epochs is 300, and the batch size is 128.

To determine the normalized values of the importance measures *I*(λ_*i*_), *i* = 1, ..., 6, we use three approaches. The first one is to apply the method called accumulated local effect (ALE) (Apley and Zhu, 2020), which describes how features influence the prediction of the black-box model on average. The second approach is to construct the linear regression model (LR) by using the generated points and their predictions obtained by means of the black-box model. The third approach is to use NAM.

The corresponding normalized values of the importance measures for λ_1_, ..., λ_6_ obtained by means of ALE, LR, and NAM are shown in Table 2. It should be noted that the importance measure *I*(λ_*i*_) can be obtained only for NAM and ALE. However, normalized coefficients of LR can be interpreted in the same way. Therefore, we consider results of these models jointly in all tables. One can see from Table 2 that all methods provide similar relationships between the importance measures *I*(λ_1_), *i* = 1, ..., 6. However, LR provides rather large values of *I*(λ_3_), *I*(λ_5_), *I*(λ_6_), which do not correspond to the zero-valued coefficients in Equation 24.

**Table 2:** Values of the importance measures in Example 1 in accordance with explanation approaches: ALE, LR, and NAM.

	**Importance measures**					
	*I*(λ_1_)	*I*(λ_2_)	*I*(λ_3_)	*I*(λ_4_)	*I*(λ_5_)	*I*(λ_6_)
ALE	0.172	0.259	0.000	0.569	0.000	0.000
LR	0.182	0.245	0.054	0.405	0.062	0.052
NAM	0.157	0.238	0.012	0.569	0.012	0.012

**Figure 7:**
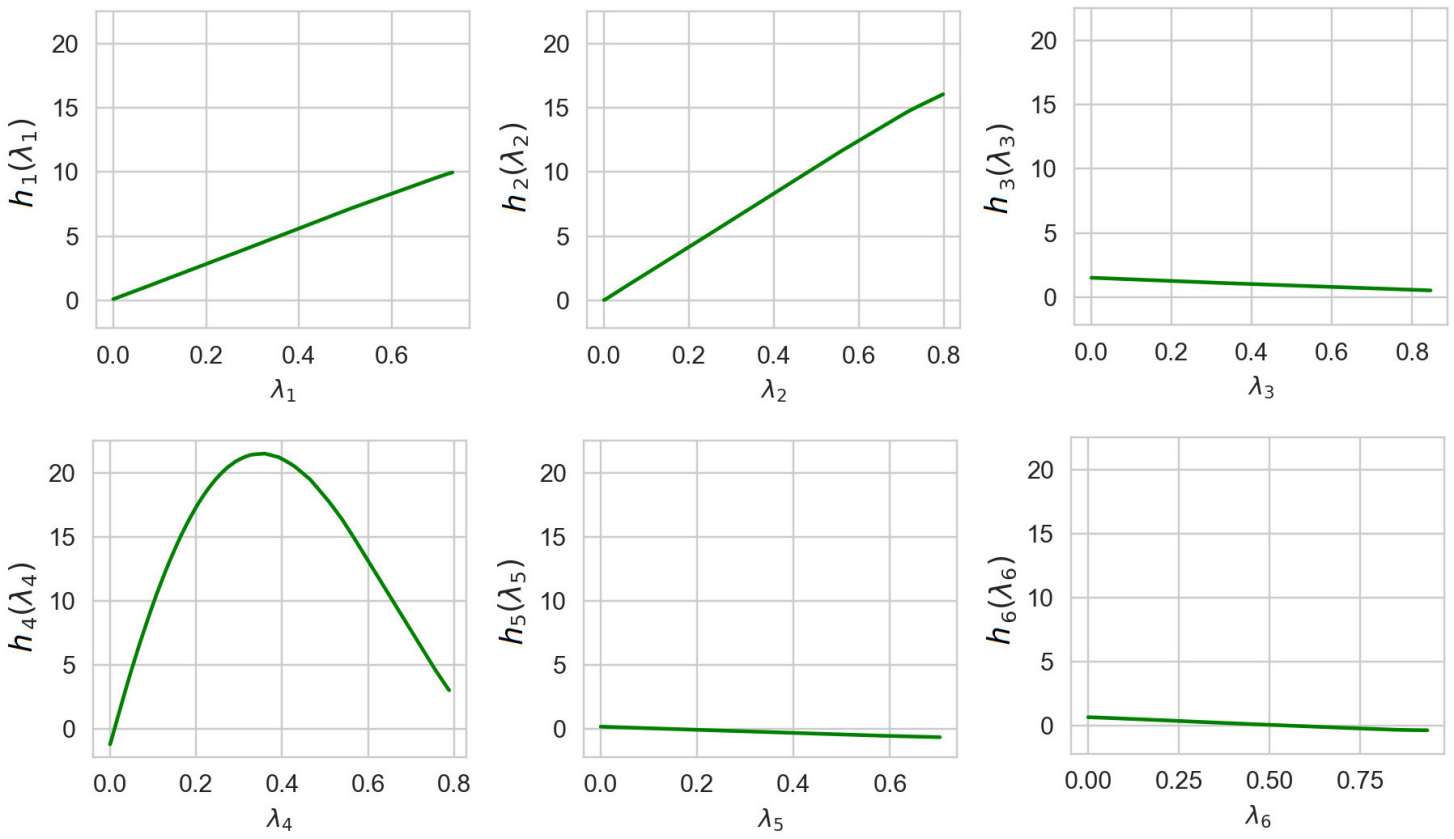
Six shape functions obtained in Example 1 for the example-based explanation.

Shape functions illustrating how functions of the generalized additive model depend on λ_*i*_ are shown in Figure 7. It can be clearly seen from Figure 7 that the largest importance λ_2_ and λ_4_ have the highest importance. This implies that the explained instance is interpreted by the fourth and the second nearest instances.

### 6.2 Example 2

Suppose that the explainable instance **x**_0_ belongs to a polytope with four vertices ${\text{x}}_{1}^{*},...,{\text{x}}_{4}^{*}$ (*d* = 4). The black-box model is a function *f*(**x**) such that


$$h\left(\text{λ}\right)={\text{λ}}_{1}^{2}+{\text{λ}}_{1}{\text{λ}}_{2}-{\text{λ}}_{3}{\text{λ}}_{4}+{\text{λ}}_{4}.$$

*n* = 1000 points λ^(*i*)^ ∈ ℝ^4^, *i* = 1, ..., *n*, are uniformly generated in the unit simplex Δ^4 − 1^. For each point λ^(*i*)^, the corresponding prediction *z*_*i*_ is computed by using the black-box function *h*(λ). NAM is trained with the learning rate 0.0005, with hyperparameter α = 10^−6^, the number of epochs is 300, and the batch size is 128.

**Figure 8:**
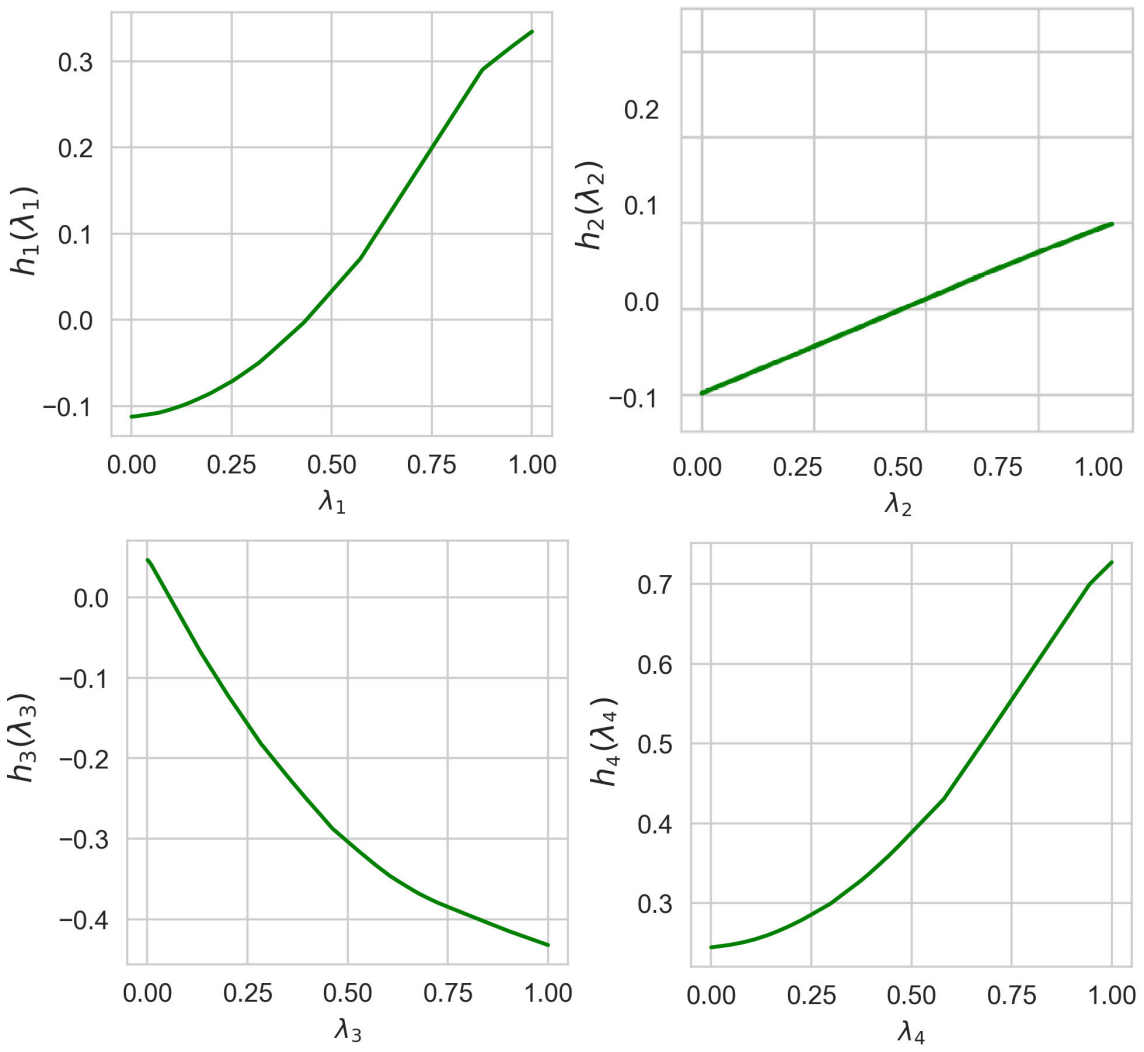
Four shape functions obtained in Example 2 for the example-based explanation.

Normalized values of *I*(λ_*i*_) obtained by means of ALE, LR, and NAM are shown in Table 3. It can be seen from Table 3 that the obtained importance measures correspond to the intuitive consideration of the expression for *h*(λ). The corresponding shape functions for all features are shown in Figure 8.

**Table 3:** Values of the importance measures in Example 2 in accordance with three explanation approaches: ALE, LR, and NAM.

	**Importance measures**			
	*I*(λ_1_)	*I*(λ_2_)	*I*(λ_3_)	*I*(λ_4_)
ALE	0.392	0.087	0.089	0.432
LR	0.357	0.081	0.112	0.450
NAM	0.306	0.134	0.202	0.358

### 6.3 Example 3

Suppose that the explained instance **x**_0_ belongs to a polytope with three vertices ${\text{x}}_{1}^{*},{\text{x}}_{2}^{*},{\text{x}}_{3}^{*}$ (*d* = 3):


$${\text{x}}_{1}^{*}={\left(-1,-1\right)}^{\text{T}},\;{\text{x}}_{2}^{*}={\left(0,2\right)}^{\text{T}},\;{\text{x}}_{3}^{*}={\left(1,0\right)}^{\text{T}}.\;$$

The black-box model has the following function of two features *x*^(1)^ and *x*^(2)^:


$$f\left(\text{x}\right)=0.7\cdot \text{sign}\left(x^{\left(1\right)}\right)+\text{sign}\left(x^{\left(2\right)}\right)$$

**Figure 9:**
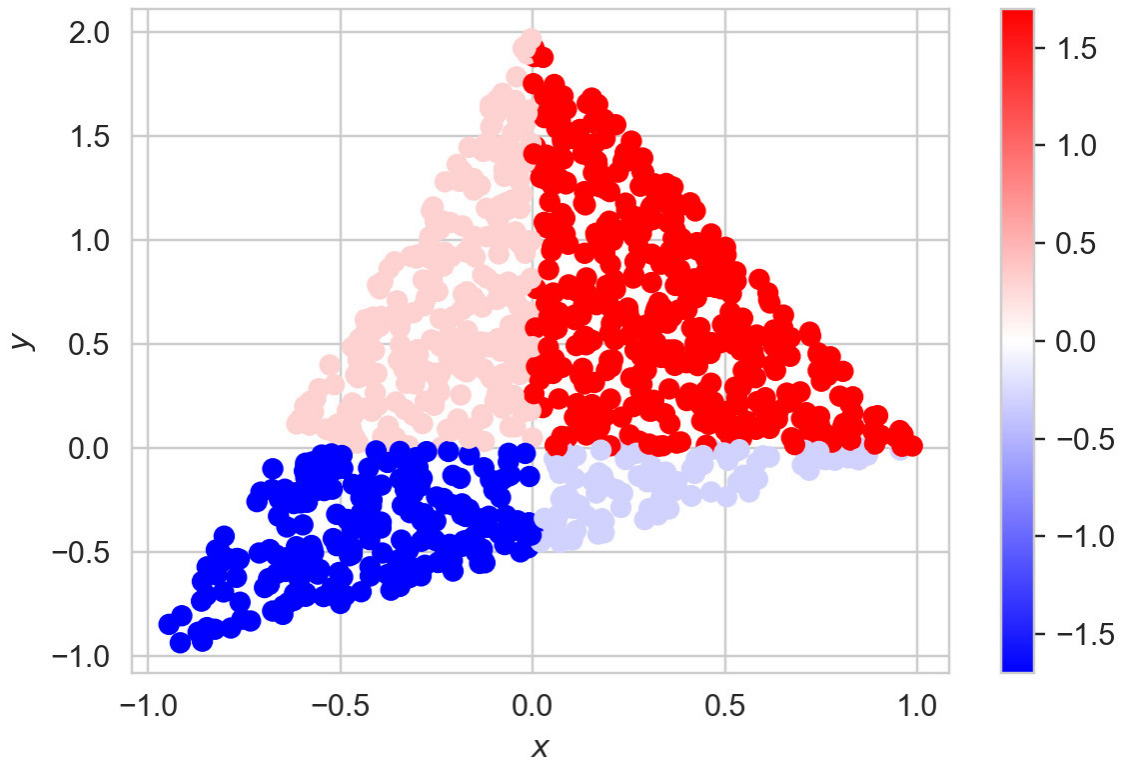
Dataset of vectors **x** and the corresponding values of *f*(**x**) for Example 3.

We generate *n* = 1, 000 points λ^(*i*)^ ∈ ℝ^3^, *i* = 1, ..., *n*, which are uniformly generated in the unit simplex Δ^3 − 1^. These points correspond to *n* vectors ${\text{x}}_{i}\in {\mathbb{R}}^{2}$ defined as


$${\text{x}}_{i}={\text{λ}}_{1}^{\left(i\right)}\cdot {\left(-1,-1\right)}^{\text{T}}+{\text{λ}}_{2}^{\left(i\right)}\cdot {\left(0,2\right)}^{\text{T}}+{\text{λ}}_{3}^{\left(i\right)}\cdot {\left(1,0\right)}^{\text{T}}$$

with the corresponding values of *f*(**x**_*i*_) and shown in Figure 9. It can be seen from Figure 9 that this example can be regarded as a classification task with four classes. Parameters of experiments are the same as in the previous examples, but α = 0.

**Figure 10:**
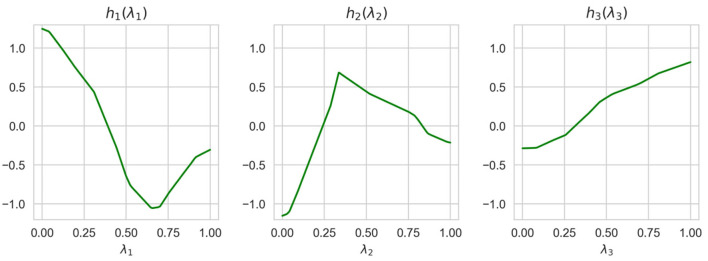
Three shape functions obtained in Example 3 for the example-based explanation.

Normalized values of *I*(λ_*i*_) obtained by means of ALE, LR, and NAM are shown in Table 4. It can be seen from Table 4 that the obtained importance measures correspond to the intuitive consideration of the expression for *h*(λ). The corresponding shape functions for all features are shown in Figure 10.

**Table 4:** Values of the importance measures in Example 3 in accordance with three explanation approaches: ALE, LR, and NAM.

	**Importance measures**		
	*I*(λ_1_)	*I*(λ_2_)	*I*(λ_3_)
ALE	0.411	0.395	0.194
LR	0.430	0.310	0.260
NAM	0.499	0.338	0.163

## 7 Discussion

Let us analyze advantages and limitations of the proposed methods. First, we consider advantages.

1. One of the important advantages is that the proposed methods allow us to replace the perturbation process of feature vectors in the Euclidean space by the uniform generation of points in the unit simplex. Indeed, the perturbation of feature vectors requires to define several parameters, including probability distributions of generation for every feature, and parameters of the distributions. The cases depicted in Figure 1 may lead to incorrect predictions and to an incorrect surrogate model. Moreover, if instances are images, then it is difficult to correctly perturb them. Due to the proposed method, the perturbation of feature vectors is avoided, and it is replaced with uniform generation in the unit simplex, which is simple. The dual approach can be applied to the feature-based explanation as well as to the example-based explanation.
2. The dual representation of data can have a smaller dimension than the initial instances. It depends on *K* nearest neighbors around the explained instance. As a result, the constructed surrogate dual model can be simpler than the model trained on the initial training set.
3. The dual approach can be also adapted to SHAP to generate the removed features in a specific way.
4. The proposed methods are flexible. We can change the size of the convex hull by changing the number *K*. It can be applied to different explanation models, for example, to LIME, SHAP, and NAM. The main idea of the adaptation is to use the well-known explanation methods. In particular, LIME can be incorporated into the proposed method by constructing the linear regression for the dual dataset. We can incorporate SHAP for computing the feature contributions of the dual instances $\left({\text{λ}}^{\left(i\right)},z_{i}\right)$. NAM is incorporated to compute the shape functions of features ${\text{λ}}_{k}^{\left(i\right)}$, *k* = 1, ..., *d*. The method can be applied to the local and global explanations. There are different definitions of the global explanation. One of the approaches to define the global explanation, proposed in Ribeiro et al. (2016), is to compute the average feature importance over the feature importances obtained by means of the local explanation for all instances of the dataset. This is a computationally difficult problem due to two main factors: (1) constructing a convex hull on the dataset; (2) solving the local explanation problems for all instances in the training set. The first problem can be solved by dividing the whole dataset into subsets with feature vectors that are close in distance construct a convex hull for each subset and solve the “local” problem of global explanation. This can be done, for example, using a decision tree so that leaves of the tree contain close instances. Another way is clustering, for which the assumption is fulfilled that each cluster also contains close instances. The second problem is computationally intensive. Its efficient solution is one of the important directions for further research.

In spite of many advantages of the dual approach, we have to note also its limitations:

1. The advantage of the smaller dimensionality in the dual representation is questionable for the feature-based explanation. If we take a number of extreme points smaller than the data dimensionality, then we restrict the set of generated primal points by some subspace of the initial feature space. This can be a reason of incorrect results. Ways to overcome this difficulty are an interesting direction for further research. However, this limitation does not impact on the example-based explanation because we actually extend the mixup method and try to find influential instances among nearest neighbors.
2. Another problem is that calculation of vertices of the largest convex hull is a computationally hard problem. This problem does not take place for the example-based explanation when the number of nearest neighbors is smaller than the initial data dimensionality.

In spite of the above limitations, the proposed approach has many interesting properties and can be regarded as the first step for developing various algorithms using dual representation. It can have the biggest impact in medicine, where, on the one hand, high-dimensional data take place, and, on the other hand, predictions (diagnoses) need to be explained to believe in them and choose a desirable treatment.

It has been shown in numerical examples with synthetic data that the proposed method outperforms the separate LIME method in terms of accuracy (see, for example, Sections 5.3, 5.4). One of the reasons is that some generated points in LIME may be located outside the training domain. However, LIME can be regarded as a part of the proposed method when it is used for computing coefficients *b* = (*b*_1_, ..., *b*_*d*_) in the dual representation. This implies that the computation time for explanation using the proposed method may exceed the LIME time. At the same time, instances in the obtained dual dataset may have the smaller dimensionality in comparison with the initial data. In this case, the computation time of the proposed method can be comparable with the LIME time.

## 8 Conclusion

Feature-based and example-based explanation methods in the framework of the dual feature representation have been presented in the study. The methods directly follow from the dual representation. They can be viewed as a basis for their improvement and the development of other methods within the dual representation.

In the example-based explanation, we used NAM as a neural network tool for explaining predictions under condition of considering the dual dataset with new variables (λ_1_, ..., λ_*d*_). However, there are effective explanation methods different from NAM, which are based on the gradient boosting machine (Nori et al., 2019; Konstantinov and Utkin, 2021). The combination of the proposed approach with these methods is an interesting direction for further research.

Another interesting direction for further research is to study how the proposed approach adapts to the example-based image explanation when *K* nearest neighbors are not determined by the proximity of original images. The search for efficient adaptation algorithms seems to be a relevant and interesting task.

There are interesting results in the linear programming when the significance of dual variables is related to perturbations of coefficients of the primal constraints (Castillo et al., 2006). This peculiarity can be applied to develop new explanation methods.

It should be noted that many applications have features that are not taken into account in the proposed approach, for example, the presence of multimodal data having different dimensions. Adaptation of the approach and the extensions oriented to specific applications are also important issues for further research. An idea behind the problem solution is to reduce different dimensions to one in the dual data representation.

Adversarial settings can produce a complex cluster structure within the feature space. A significant challenge in such scenarios is addressing out-of-distribution points. The proposed method can handle this problem unlike the LIME. To enhance the robustness, we propose two hyperparameters: the configuration of the Dirichlet distribution and the number of the neighbors to construct the convex hull. Proper adjustment of these hyperparameters has the potential to enhance the method's robustness.

The proposed results are fundamental. They are illustrated only with synthetic data or well-known real datasets. Therefore, we do not use personal data which require to implement robust security measures to safeguard sensitive information and prevent unauthorized access. It should be noted that one of the important goals of the proposed results is to provide explanations for the machine learning model decisions and actions making the models transparent. As a result, users have a clear understanding of how the black-box model operates and the factors influencing its outputs. The proposed method belongs to the field of explainable artificial intelligence; thus, we have contributed to the development of transparent and reliable AI systems. Methods of the prediction explanation can improve collaboration between AI developers and domain experts as they can be used to facilitate the feedback exchange between the AI engineer and the expert. Our method can be more useful in domains where the example-based explanations are in demand. The potential risks and biases associated with the proposed method are comparable to those of the LIME method and depend on the data scientist's handling of the data.

## Data Availability

Publicly available datasets were analyzed in this study. This data can be found here: https://archive.ics.uci.edu/dataset/294/combined+cycle+power+plant.
